# Drug-drug interactions and risk of bleeding among inpatients on warfarin therapy: a prospective observational study

**DOI:** 10.1186/1477-9560-12-20

**Published:** 2014-09-17

**Authors:** Gebrehiwot Teklay, Nuredin Shiferaw, Befikadu Legesse, Mebratu Legesse Bekele

**Affiliations:** 1 Department of Pharmacy, College of Health Sciences, Mekelle University, P.O. Box: 1871, Mekelle, Ethiopia; 2 School of Medicine, College of Medicine and Health Sciences, Wolaita Sodo University, Wolaita Sodo, Ethiopia

**Keywords:** Drug-drug interaction, Warfarin, Bleeding, Ethiopia

## Abstract

**Background:**

Warfarin is known for its interaction with many drugs, resulting in undesired treatment outcomes such as bleeding. The study aimed to assess the prevalence of drug-drug interactions and determinants of bleeding among inpatients on warfarin therapy.

**Methods:**

A cohort of inpatients on warfarin treatment was prospectively followed from date of admission until discharge. The study was carried out from January to October 2013 in Ayder Referral Hospital, Northern Ethiopia. Patients on warfarin therapy during the study period and willing to participate were included as study subjects. Each concurrent medication was collected and checked for drug-drug interactions using Micromedex® online drug reference. Data were analyzed using statistical software, SPSS for windows version 16. The relationship between bleeding complications and independent variables (age, sex, residence, type and number of co-medications, dose and duration of warfarin treatment, INR value) was assessed using binary logistic regression analysis (Odds ratio, 95% confidence interval).

**Results:**

Of the total 133 patients enrolled in the study, 78 (58.9%) were females. The mean age of the study participants was 40.81 ± 17.6 years. The prevalence of drug-drug interactions was 99.2%. Among these, 65 (49.2%) patients had at least one major while the others had moderate level of drug-drug interaction. Twenty two (16.5%) patients have developed bleeding complications. Increase in international normalized ratio value was found to be strongly associated with risk of bleeding (P value = 0.00; OR = 0.03 (0.00-0.46)).

**Conclusion:**

Drug-drug interactions with warfarin were prevalent in the study hospital. Bleeding complications due to warfarin were also high. Thus, clinicians should be aware of potential interactions and monitor patients’ international normalized ratio closely.

## Background

Cardiovascular diseases are the most common causes of death worldwide [1]. The burden is growing in countries like Ethiopia, previously assumed to be with low prevalence [2]. Warfarin, a medication that inhibits the synthesis of clotting factors, is the most commonly used oral anticoagulant for the prevention and treatment of various cardiovascular disorders [3-7].

Warfarin is known for its variable dose–response relationship, narrow therapeutic index, potential bleeding risk and the potential for numerous drug and dietary interactions [8]. Monitoring the international normalized ratio (INR), a measure of warfarin’s effect on clotting factors and the blood’s propensity to clot, is therefore essential for maintaining the drug within its narrow therapeutic window of 2.0–3.0 [6,9]. Maintaining the target INR is essential for patient safety. Below-target INR is associated with under anticoagulation, whereas above-target INR leads to hemorrhagic complications [8,10-13]. Hemorrhage is a concern particularly when warfarin is used concomitantly with other interacting drugs. Several factors may increase the risk of over-anticoagulation and bleeding; drug interactions usually account for the majority of the risk [4,13].

This study is particularly important in resource limited countries like Ethiopia. There is increasing burden of non communicable diseases on top of the existing infectious diseases such as tuberculosis and HIV/AIDS [2,14]. Most of these chronic diseases require multiple medications and prescribers in such setting are overburdened [15]. There is less emphasis of physicians on drug interaction and polypharmacy [16,17]. To the best of available literature, no research has been done in Ethiopia related to warfarin drug-drug interactions (DDIs). This study was aimed to assess the prevalence of DDI and determinants of bleeding among inpatients on warfarin therapy in Ayder Referral Hospital.

## Methods

This study was conducted in the internal medicine ward of Ayder Referral Hospital situated in Mekelle town, northern Ethiopia. The hospital started giving referral and specialized medical services in 2008 to about 8 million inhabitants in its catchment area of northern Ethiopia. It provides a broad range of medical services to both in and outpatients.

A prospective observational study was carried out on a cohort of patients on warfarin to investigate DDIs and determinants of bleeding. Patients on warfarin therapy, admitted to the internal medicine ward, from January to October, 2013 were followed from the time of admission until discharge. The source population was all inpatients treated with warfarin in Ayder Referral Hospital. Patients on warfarin therapy and willing to participate after informed consent were included as study subjects. Patients were included in the study regardless of age, sex, severity of disease, type of disease, duration of warfarin therapy and type of co-medication.

The sample size required for the study was determined using Epi-Info; considering the prevalence of drug-drug interaction 84% (16); 5% margin of error at 95% confidence level and finally adjusting for finite population correction a sample of 133 patients was taken. Systematic random sampling (sampling interval (k) of N/n (500/133 = 4)) was used to enroll participants from the source population. The study was conducted after ethical approval was obtained from ethics review committee of College of Health Sciences, Mekelle University.

Data were collected by two pharmacists trained particularly for this study. Information about patient demographics (age, sex, residence), warfarin indication, co-morbidities, warfarin dosing and duration of treatment, concomitant drugs, sign and symptoms of gastrointestinal bleeding, and INR values were collected from the patient, treatment charts, and laboratory notes.

Micromedex® online drug reference [18], software was used to screen patients for DDIs. All drugs in a patient’s medication profile were entered one by one into the software. The software display(s) all interacting combination(s) present in the medication profile. It also provides information about the mechanism and potential adverse outcomes of an interaction. Except some modifications (additions) by the authors, definitions below are adopted from Micromedex®.

***Drug-drug interaction (DDIs)*:** Drug-drug interaction is the alteration of a drug’s pharmacologic or clinical response by co-administered drug. Drug interaction can be of minor, moderate and major type.

***Moderate drug interaction***: A type of drug interaction that may cause deterioration of a patient’s clinical status, requiring additional treatment, hospitalization or extension of hospital stay. This needs close monitoring of the patient. It may necessitate discontinuation of treatment.

***Major drug interaction***: A type of potentially life threatening interaction, capable of causing permanent damage, and necessitating additional treatment, hospitalization or extension of hospital stay. Such interaction necessitates discontinuation of the treatment.

***Clinically significant DDIs***: Drug-drug interactions resulted in clinically observable response (example: bleeding and/or change in INR for warfarin).

### Statistical analysis

Study variables were coded (from VR001 to VR026) and entered into Epi info version 7. Then, data were transferred into statistical software, SPSS for windows version 16. Descriptive statistics was used to describe number and percentages, mean and standard deviations. The relationship between bleeding complications and independent variables (age, sex, residence, type and number of co-medications, dose and duration of warfarin treatment, INR value) was assessed by multivariate binary logistic regression. Backward elimination (likelihood ratio) was used as variable selection method. Estimates of risk factor were expressed as odds ratio (OR), at 95% confidence interval (CI). P value of less than 0.05 was considered significant.

## Results

Of the total 133 patients enrolled in the study, 55 (41.1%) were males and 78 (58.9%) were females. The mean age was 40.8 ± 17.6 and majority of the participants (54.3%) were between age 15 to 39. Seventy seven (57.9%) of the studied patients were urban residents (Table 1).

**Table 1:** Demographic characteristics of patients

**Characteristic**		**Number (%)**
Sex	Male	55 (41.4%)
	Female	78 (58.6%)
	Total	133
Age category (years)	15-39	73 (54.3%)
	40-64	44 (33.1%)
	≥65	16 (12.6%)
	Total	133
	Mean	40.8
	Standard deviation	17.6
Residence	Urban	77 (57.9%).
	Rural	56 (42.1%)
	Total	133

**Figure 1:**
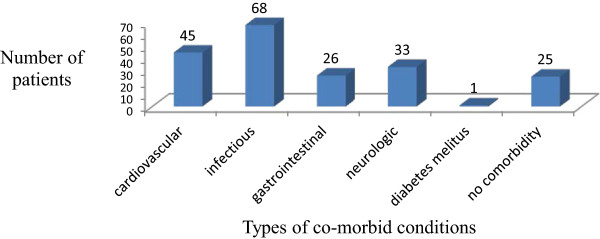
Types of co-morbid conditions among patients on warfarin therapy.

There were 6.0 ± 3.3 mean number of medications prescribed per patient at the time of screening for drug interaction. The most common indication for warfarin therapy in this population was for prevention and treatment of deep vein thrombosis (61.7%) followed by atrial fibrillation (21%). Infectous and cardiovascular diseases were the common type of commorbidities (Figure 1). Majority of the patients (58.6) were using 5 mg warfarin. Duration of warfarin therapy was considered with respect to use prior to admission and the time of hospital stay as inpatient. Fifty seven (42.9%) of the patients were on warfarin for more than three months while the remaining 76 (57.1%) patients were on warfarin for less three months (Table 2).

**Table 2:** Treatment characteristics of patients

**Characteristic**		**Frequency**	**Percent**
Warfarin indication	Prevention and treatment of deep vein thrombosis	82	61.7
	Pulmonary embolism	13	9.8
	Atrial fibrillation ± Stroke	28	21
	Myocardial infarction	6	4.5
	Unspecified	4	3.0
Number of drugs per patient	<3	26	19.5
	3-5	35	26.3
	6-8	40	30.1
	>8	32	24.1
	Mean	6.0	
	Standard deviation	3.3	
Duration on warfarin therapy	< 1 month	20	15.0
	1-3 months	56	42.1
	>3 months	57	42.9
	Mean	2.2	
	Standard deviation	0.7	
Warfarin dose^^^	2.5 mg	25	18.8
	5 mg	78	58.6
	7.5 mg	22	16.7
	10 mg	5	3.8
	≥12.5 mg	3	2.3
No of interactions per patient	0	1	0.8
	1	31	23.2
	2-4	71	53.4
	≥5	30	22.6
	Total	133	100
	Mean	3.2	
	Standard deviation	2.1	

^^^Dose at the time of screening for drug interaction and bleeding.

**Figure 2:**
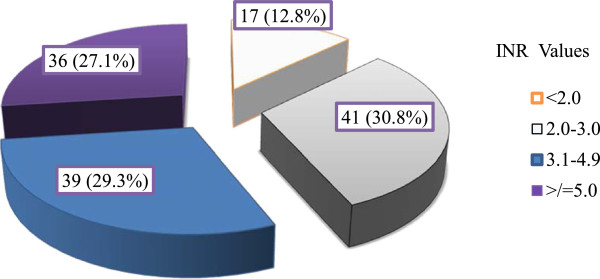
INR values of patients on warfarin therapy.

According to the Micromedex® online drug reference, a total of 428 DDIs with warfarin were identified. One hundred thirty two (99.2%) patients had at least one drug-drug interaction. There were 3.2 ± 2.0 mean number of DDIs per patient. The most common type of interaction was moderate type which accounted for 72.4% while the rest 27.6% were major type of interaction. Sixty seven (50.8%) patients had moderate while, 65 (49.2%) patients had at least major DDI. The most frequent interacting drugs were antibiotics 158 (36.9%), followed by anticoagulants (heparin and enoxaparine) 102 (23.8%), Cardiovascular drugs (spironolactone 33 (7.71%) and β-blockers 12 (2.8%)), acid suppressing agents 34 (7.94%), aspirin 20 (4.67%) and Non-steroidal anti-inflammatory drugs (NSAIDs) 10 (2.3%) (Table 3).Of all patients studied, only 30.8% were on the target INR (2.0-3.0) at the time of evaluation for drug interaction and bleeding; while 12.8% and 56.4% had INR value below 2.0 and greater than 3.0 respectively (Figure 2). Gastrointestinal bleeding was seen in 22 (16.5%) patients. Half (50.0%) of the bleeding complications occurred in the age group of 15 to 39. Twelve (54.5%) patients had moderate DDIs while 10 (45.5%) of them had major type DDIs. Among patients with bleeding, 20 (90.9%) patients had INR value of >5. Vitamin K was administered along with temporarily or permanent discontinuation of warfarin for the management of observed bleeding.

**Table 3:** List of drugs interacting with warfarin

**Drug class**	**Drugs**	**No. of patients**	**Level of interaction**	**Drug class**	**Drugs**	**No. of patients**	**Level of interaction**
Antibiotics	Ceftriaxone	30	Moderate	Anticoagulants	Heparin	101	Moderate
	Cotrimoxazole	22	Major		Enoxaparine	1	Major
	Nevirapine	14	Moderate				
	Clarithromycin	11	Major	Cardiovascular	Spironolactone	33	Moderate
	Rifampin,	18	Moderate		Atenolol	9	Moderate
	Isoniazid	18	Moderate		Propranolol	3	Moderate
	Norfloxacin,	8	Major		Amiodarone	1	Major
	Benzathine penicillin	8	Major				
	Ciprofloxacin	6	Major	Acid suppressing agents	Omeprazole	18	Moderate
					Cimetidine	15	Moderate
	Amoxicillin	4	Major		Pantoprazole	1	Moderate
	Metronidazole	3	Major				
	Ceftazidime	2	Major				
	Cephalexin	2	Major				
	Cloxacillin	2	Major	Analgesics	Aspirin	20	Major
	Vancomycin	1	Moderate		Diclofenac	9	Moderate
	Erythromycin	1	Major		Ibuprofen	1	Moderate
	Cefotaxime	1	Major		Acetaminophen	2	Moderate
	Miconazole	6	Major		Tramadol	20	Moderate
	Fluconazole	1	Major				
				Corticosteroids	Prednisolone	3	Moderate
					Dexamethasone	2	Moderate
					Hydrocortisone	1	Moderate
Lipid lowering	Simvastatin	20	Major	Others	Amitriptyline	2	Moderate
					Propylthiouracil	1	Moderate
					Glibenclamide	1	Moderate
Anti-epileptic	Phenobarbitone	2	Moderate		Lactulose	1	Major
	Na valproate	2	Major				
	Phenytoin	1	Moderate				

Multivariate binary logistic regression analysis was used to assess factors associated with bleeding complications. Among the variables investigated, only INR value was found statistically significant association with bleeding complications (P value = 0.00; OR = 0.03 (0.00-0.46)). The effect of other variables including interacting drugs was not statistically significant (Table 4).

**Table 4:** Multivariate binary logistic regression analysis of factors associated with bleeding complications

**Characteristics**		**Bleeding**		**Multivariate**	
		Yes	No	P-value	OR (95% CI)
Sex	Male	11	43		1
	Female	11	67	0.38	2.13 (0.38-11.82)
Residence	Urban	14	63		1
	Rural	8	48	0.15	3.76 (0.60-23.47)
Age	15-39	11	62		1
	40-64	8	36	0.75	0.72 (0.09-5.49)
	65 and above	3	13	0.07	0.08 (0.05-1.31)
Warfarin dose	2.5 mg	3	22		1
	5 mg	14	64	0.44	0.40 (0.04-4.15)
	7.5 mg	3	19	0.77	1.63 (0.06-43.75)
	≥10 mg	2	6	0.20	0.11 (0.00-3.19)
Duration of treatment	<1 month	2	18		1
	1-3 months	8	49	0.83	1.39 (0.07-27.99)
	>3 month	12	44	0.66	1.98 (0.09-42.18)
Number of drugs	<3	6	20		1
	3-5	8	27	0.99	1.03 (0.02-55.25)
	6-8	4	36	0.16	2.7 (0.68-10.71)
	>8	4	28	0.29	2.10 (0.52-8.42)
Number of interactions	<2	7	25		1
	2-4	9	62	0.92	0.82 (0.02-41.11)
	≥5	6	24	0.24	0.05 (0.00-7.13)
Type of interaction	Moderate	13	54		1
	Major	9	56	0.39	1.52 (0.60-3.80)
INR value	<5	2	95		1
	≥5	20	16	0.00*	0.03 (0.00-0.46)

*Statistically significant association.

## Discussion

In this study a patient was prescribed a mean number of 6.0 ± 3.3 medications, and there were 3.2 ± 2.0 drug-drug interactions per patient. The overall prevalence of DDIs was 99.2%. About half of these interactions were clinically significant resulting in change in INR and/or bleeding. Parallel to this study, an interventional study on recognition and management of drug*–*drug interactions in inpatients at the Cantonal Hospital of Baden, found 567 DDIs in 502 inpatients, of which 419 (74%) were judged to be clinically relevant [19]. Likewise, the prevalence of DDIs in outpatients was reported as high as 84% [20,21]. Higher prevalence of DDIs is expected from inpatients as compared to outpatients as more intensive management protocols are required to manage inpatients. Inpatients usually have multiple co-morbidities requiring many medications while outpatients are relatively stabilized. It might also be due to the limited options physicians had to treat a condition using non-interacting drugs which is a common problem in developing countries. Trend of medical practice, prescribers’ knowledge and practice and absence of clinical pharmacists who have role in recognition and management of drug interaction might also contribute.

The prevalence of venous thromboembolism is strongly age-related, increasing nearly 90 fold from <15 to >80 years old [22]. In the developed world (Europe and USA) anticouglation therapy is commonly used for elderly patients [3,9,19,21]. In contrast, in this study above half of the subjects on warfarin were in the age less than forty. In developing countries the burden of serious infections such as HIV and tuberculosis is affecting the young adults (age <40) and making patients to stay bed redden for longer time. Such patients require prophylactic anticoagulation and in this study warfarin was commonly indicated for prevention and treatment of deep vein thrombosis. Rheumatic heart disease, hypertension and venous thrombosis were previously reported as the common types of cardiovascular diseases in Ethiopians, and affecting the young adults [23,24]. There are some reports of increasing physical inactivity, alcohol use, khat use, smoking and change in dietary habits among young adult Ethiopians [2,25]. Awareness, treatment and control of hypertension and other cardiovascular risk factors are very poor in the Ethiopian settings [2]. This may lead to development of cardiovascular diseases such as stroke and myocardial infarction at early age. But further studies are needed to investigate the increase in cardiovascular diseases among young adult Ethiopians.

Commonly co-prescribed drugs found interacting with warfarin were antibiotics (ciprofloxacin, norfloxacin, clarithromycin, cotrimoxazole, amoxicillin, cloxacillin, cephalosporins, erythromycin, metronidazole, ceftriaxone, rifampin, isoniazid, and nevirapine). The finding of this study is consistent with previous studies [26-29]. One analysis in Norway showed that heparin, antibacterial and NSAIDs were common interacting drugs [26]. In parallel a retrospective cohort study, oral antibiotics ((azithromycin, levofloxacin, and Trimethoprim/sulphamethoxazole (TMP/SMX) were found to increase the incidence and degree of over anticoagulation [28]. Except rifampicin, most antibiotics are liver enzyme inhibitors and their interaction with warfarin may contribute to over anticoagulation.

Corticosteroids and NSAIDs were the other classes of drugs found to interact with warfarin. This finding is consistent with study by Kotirum, et al. [20] and other studies from different parts of the world [9,21,26,29-33]. Understanding the severity of the drug interaction is important for practitioners because major type of interaction is more likely to produce negative outcomes which include either ineffectiveness or over anticoagulation and bleeding risk. According to this study about 49.2% patients had at least major type DDIs while 50.8% had moderate DDIs.

The nearest INR value at the time of screening for drug interaction and bleeding was considered as indicative of coagulation status. Most of the patients did not achieve their target INR values. Only 41 (30.8%) had an INR value of 2.0-3.0; the remaining 17 (12.8%), 75 (56.4%) patients had <2.0 and >3.0 INR value respectively. This result showed that in 69.7% of patients a target INR value was not obtained. The percentage of supratherapeutic in this study was much higher than a study by Verhovsek et al. [10]; the reported INR value within, below and above the therapeutic range were 54%, 35% and 11%, respectively. But this study took mean INR value of the patients while in this study the nearest INR value at the time of screening for drug interaction was considered. In addition, the higher number of patients with supratherapeutic values in the current study might be due to higher prevalence of DDIs and lack of frequent INR monitoring in such resource limited setting.

In this study prevalence of gastrointestinal bleeding was found to be 16.5% which is near to a study carried out by Zhang et al. [3], which reported a prevalence of 14.7%. But it was lower than that reported by Meegaard et al. [33]; 22% of the patients had a verified bleeding episode. This higher percentage might be due to inclusion of only those high risk patients with INR level greater than 6.5 or INR of 3.5 and above. Multivariate binary regression analysis of variables indicates that, INR value was statistically associated with bleeding. As compared to patients with INR value less than five, patients with INR value of greater than 5.0 were 33 times higher at risk of bleeding [OR = 0.03 (0.00-0.46)]. This finding is in line with a recent Norwegian study reported that 74% of patients treated with warfarin had INR values above the therapeutic range at the time of gastrointestinal bleeding [34]. Other factors such as age, concurrent drug use, dose, and duration of warfarin treatment were not significantly associated with bleeding complications in this study population.

Even though none of the observed drug-interactions in this study had statistically significant association with risk of bleeding many other studies reported NSADs interacting with warfarin associated with increased risk of serious bleeding [8,21,34-38]. For instance, the combined use of warfarin and aspirin vs. warfarin alone was 4.5 (95% CI: 1.1–18.1) in a study by Gasse et al. [8]. Likewise, there were a 13-fold (95% confidence interval, 6.3 to 25.7) increase in the risk of developing hemorrhagic peptic ulcer disease in concurrent users of oral anticoagulants and NSAIDs in another study [38]. Some antibiotics were also reported to increase risk of warfarin bleeding. Fischer et al. [39] found ciprofloxacin and cotrimoxazole associated with increased risk of upper gastrointestinal tract hemorrhage. Compared with the use of warfarin alone, the use of either cephalosporins (OR 1.157; 95% CI, 1.043-1.285) or metronidazole (OR 1.578; 95% CI, 1.321-1.886) were associated with increased risk of hemorrhage, whereas the risk of hemorrhage was not greater with concomitant use of NSAIDs as reported by Zhang et al. [3]. In this study, the small sample size as compared to the above studies might have contributed for the lack of statistically significant association between the type of drug interaction and observed treatment outcomes.

## Conclusion

In this study, drug-drug interactions were prevalent. Commonly co-prescribed drugs interacting with warfarin were antibiotics, anticoagulant, diuretics and NSAIDs. Bleeding complications were significantly associated with increased INR value. Clinicians should give attention to potential drug interaction while prescribing drugs in patients with warfarin. Frequent monitoring of INR value is vital to predict treatment outcome of patients on warfarin. Patients should also be counseled about drug interactions, sign and symptoms of warfarin related bleeding complications.

## Abbreviations

CI: Confidence interval; DDIs: Drug-drug interactions; HIV/AIDS: Human immunodeficiency virus/acquired immunodeficiency syndrome; INR: International normalized ratio; NSAIDs: Non-steroidal anti-inflammatory drugs; OR: Odds ratio; TMP/SMX: Trimethoprim/Sulphamethoxazole; VR: Variable.

## Competing interests

The authors declare that they have no competing interests.

## Authors' contributions

GT and NS contributed to study design, data collection, data analysis and developed of the draft manuscript. BL and MLB assisted with data analysis, interpretation of finding and revision of the manuscript. All authors have read and approved the final manuscript.

## Authors' information

GT is a lecturer and clinical pharmacist in Mekelle University, College of Health Sciences; BL is a Lecturer of pharmacoepidemiology and social pharmacy in Mekelle University, College of Health Sciences; MLB is a lecturer and clinical pharmacist in Wolaita Sodo University, College of Medicine and Health Sciences; NS is working as Hospital pharmacist.
